# Observations on Intra-Cranial Tuberculosis in Childhood

**Published:** 1907-08-24

**Authors:** William P. S. Branson

**Affiliations:** Assistant Physician, East London Hospital for Children


### OBSERVATIONS ON INTRA-CRANIAL TUBERCULOSIS IN CHILDHOOD.

By WILLIAM P. S. BRANSON, M.D., M.R.C.P., Assistant Physician, East London Hospital for Children.

Intra-cranial tuberculosis is always secondary to an infected focus elsewhere, and during childhood supplies the closing scene in a majority of tuberculous deaths. Therefore every tuberculous lesion, however insignificant intrinsically, is a potential danger to life, especially in the early years of childhood, when caseation and subsequent dissemination of the tubercle bacillus are such common incidents of tuberculosis.

There are three main classes of lesion, each of which has for a time a more or less distinct clinical representation, though all merge into a common type towards the end of life. These are:—

1. Miliary meningeal tuberculosis,
2. Caseating meningitis,
3. Caseous tumour of the brain.

### 1. Miliary Meningeal Tuberculosis.

This is the common form. It is marked by thickening and adhesion of the membranes about the base and by small tubercles in varying abundance. The vertex is often free from macroscopic tubercles after death, but there is no rule except that the base is the region principally involved. I have made some inquiries into the seat of the primary infection in these cases, confining attention to those patients who were believed to be free from tuberculous lesions at the time of the appearance of the meningeal symptoms. This appears to be almost invariably a gland; the bronchial lymphatic glands, being caseous in about 50 per cent. of instances, are the chief offenders. In about 20 per cent. the mesenteric glands appear to be responsible.

### The Symptoms.

These are best studied in cases where the meningeal affection is clinically primary—cases, that is to say, where the original focus is latent in the lymphatic glands, as above described—for in them the symptoms are not veiled by those of disease elsewhere. The cardinal symptoms of onset in such patients as these are, in order of frequency, vomiting, convulsions, and headache. Vomiting is essentially an early symptom, and is often very frequently repeated during the first week. At or about this time it ceases and generally plays no part in the later stages of the malady; but there may be no vomiting throughout. Convulsions may assume all forms, from a limited Jacksonian seizure without loss of consciousness, to a long-continued generalised fit. These motor invasions may pass away, leaving no trace of illness behind, and thus constantly imperil the diagnosis. Headache is probably more constant than records appear to show, for it is liable to be overlooked in young children. It is often very severe and subject to paroxysmal reinforcement, which seems to determine the shrill "meningeal cry."

In addition to these three cardinal symptoms there are two others as constant but less dramatic. These are irritable drowsiness and constipation. The former is common at the invasion of any acute illness in childhood, but is generally associated with a considerable rise of temperature. When it appears apart from high fever it is an ominous symptom. Constipation is almost constant during the invasion of tuberculous meningitis.

The medullary irritation which may be presumed to account for the early vomiting may, and generally does, affect to a varying extent the cardio-inhibitory, the respiratory, and the vasomotor centres. In consequence it is common to meet with irregularities of the pulse and respiration, together with the *tâche cérébrale.* Of these the two first are of serious moment, but the last has no specific significance. Yet another important symptom is an unnatural flaccidity of the abdomen, which is frequently to be observed in the early stages of the disease. It is not easy to explain the mechanism of this symptom, which is a very common one. Photophobia is common, but examination of the fundus is of less diagnostic service than is commonly reported. For although both optic neuritis and choroidal tubercles are to be met with tolerably often, they do not as a rule appear early enough to assist the diagnosis. Kernig's sign occurs in so many conditions as to be almost valueless.

The duration of life after the onset of symptoms varied in a series of thirty-seven cases investigated from three to twenty-six days.

### The Diagnosis.

It is well known that the diagnosis of tuberculous meningitis is full of pitfalls, particularly because it often attacks children apparently in the best of health. The points for insistence are that the age of election for apparently primary tuberculous meningitis is the second year of life, and that the disease becomes progressively less frequent in after years; also that the most common error lies in attributing the vomiting of onset to simple "biliousness"—that is to some purely gastric cause. It is not too much to say that the appearance in a child under five years old of any one of the above-mentioned symptoms should bring the possibility of tuberculous meningitis to mind. It should be remembered, further, that, with the exception of rare cases of unaccountable vomiting, all the diseases commonly confounded with tuberculous meningitis present either a considerable degree of fever, or a leucocytosis, or both, whereas the fever of tuberculous meningitis is trifling and leucocytosis absent at least in the early stages.

### Prognosis and Treatment.

The fact that a few cases of recovery from what appears to have been miliary meningeal tuberculosis are to be found in the literature does not appreciably vitiate the general dogma that the disease is a mortal one. But it justifies the assumption that there is a bare chance of recovery which should never be denied to the parents of the victim. In view of the sad facts of its pathology and probable issue, it is hard to approach the treatment of this disease with any enthusiasm, but there is a definite opening for therapeutics in the relief of symptoms. This is particularly true of the headache which attends the early stages. I have no doubt that both phenacetin and antipyrin are of definite service here. Further, it is good practice to shave the head and advise the constant application of ice, for the proceeding may give some relief, and at the worst supplies an active outlet for maternal solicitude, which is not without its value in such gloomy circumstances. If these means do not affect relief of the pain, opium or morphine should certainly be given. Convulsions in the early stages should be met by chloroform inhalations, but the clonic twitchings which attend the close of the disease are past all useful interference.

*(To be concluded.)*

